# Utilization of dental care service and associated factors among pre-school children in northwest China over the past decade

**DOI:** 10.1186/s12903-023-02736-2

**Published:** 2023-01-30

**Authors:** Xiao Hu, Xiaoyu Fan, Jiangang Tian, Bin Zhang, Ruizhe Huang

**Affiliations:** 1 Key Laboratory of Shaanxi Province for Craniofacial Precision Medicine Research, College of Stomatology, Xi’an Jiaotong University, Xi’an, China; 2 Clinical Research Center of Shaanxi Province for Dental and Maxillofacial Disease, College of Stomatology, Xi’an Jiaotong University, Xi’an, China

**Keywords:** Pre-school children, Utilization, Dental care, Northwest China, Caregivers

## Abstract

**Objective:**

The purpose of this study was to analyze the factors influencing the utilization of oral health care among 5 years old children.

**Methods:**

We conducted two observational cross-sectional studies. The studies were conducted in 2005 and 2015 and included 5-year-old children who underwent dental examination by trained dentists and the caregivers of the children were requested to answer the questionnaire. Multi-level stratified sampling method was used. Chi-square tests were used to analyze the utilization of dental care and other socio-economic variables. Logistic regression models were employed to explore the primary factors influencing the use of dental care among pre-school children.

**Results:**

In 2005, a total of 399 and in 2015, 492 child-caregiver pairs were included. The majority of the caregivers in both surveys were females, comprising 68.2% and 74.8% of the caregivers in 2005 and 2015, respectively. 75.2% and 87.0% (*p* < 0.05) of the respondents had an education level of lesser than 9 years. The prevalence of caries was higher in 2015 (63.2%) (*p* < 0.05) than in 2005 (53.4%). In 2005 and 2015, the utilization of dental care services was 20.8% and 20.0%, respectively. A statistically significant association was observed between caries and dental care use in 5-year-olds over the past decade. After adjusting for confounders, dental service usage among children in urban areas was 1.62 times higher than that of rural areas in 2005 (95% CI 0.069–0.571), and the self-assessment of caregivers regarding their child’s oral health significantly improved oral health use in 2015.

**Conclusion:**

The utilization of dental care services over the past decade is insufficient among pre-school children in northwest China. Hence, with the decreasing gap about economic and health service resources, policymakers should place greater emphasis on raising awareness among caregivers about the oral health status of their children.

## Introduction

China has launched various programs to promote dental health among children ranging from “happy mouth, happy family” organized by the national health commission to comprehensive intervention programs for deciduous caries in pre-school children [1, 2]. While pre-school children oral health status is still a serious global public health problem. For instance, Meng’s study shows that among children oral pathologies continue to be a significant health issue [3]. A study has shown that a large proportion of 5-year-old children experience oral mucosal lesions [4]. Children living in rural areas suffer from higher rates of caries and oral health diseases [5]. The prevalence of caries among 5 years old children has increased from 66.0% in 2005 to 71.9% in 2015 in China [6].

Oral health services comprise an integral part of public health services. Understanding the factors influencing utilization of children's oral health services can assist in rationally allocating the resources and hence dramatically improving the social and economic benefits. Children with severe dental caries predominantly seek dental care services [7], while a child is not the decision-maker whether to consult a dentist or not [8]. Studies have revealed that parents or caregivers play an important role in children’s oral health. Their socio-economic status, knowledge, and attitude toward oral health are significantly associated with children’s oral health status [9–11]. For a low-income family, high quality of primary health care attributes in dental services use [12, 13].The study by Ou et al. [14] has reported that the number of parents actively seeking dental care for their children is low, and the primary purpose for consultation was treatment rather than prevention. The study conducted by Meng et al. [3] also demonstrated that the rate of seeking dental care was low and that of self-medication was relatively high.

Caregivers are undoubtedly an important predictor of dental health services utilization among their children [15, 16]. Caregivers with higher education and urban residents are positive determinants to better outcome [17]. Higher socioeconomic groups, better family oral health practices and better knowledge are beneficial for children and contribute to access to improved dental care [18, 19]. On the contrary, with the recent economic advancement in China, lack of understanding of caregivers about their child’s oral health status contributes to the lower utilization of dental care services [20–22].

The purpose of this study is to describe the oral health status and to identify the type of the children’s dental care utilization over the past decade. We also explored whether the association of caregiver’s perception of their child’s oral health status and the dental care utilization has changed in northwest China.

## Methods

### Study population and design

According to the oral health epidemiological investigation protocol recommended by WHO, we conducted two observational cross-sectional studies. The studies were conducted in 2005 and 2015 among pre-school children and their caregivers in Shaanxi province, the northwestern part of China. The sampling was done by complex probability sampling design in three steps. In the first step, we used PPS (probability proportionate to size sampling) to choose two districts and two counties. In the second step, we used the same design to select three kindergartens. In the third step, we have chosen 5-years old children from selected schools.

The inclusion criteria were: age 5 years, resident time in the local area > 6 months,informed consent of parents. The exclusion criteria werethose who were not cooperative during the study.

### Data collection and measures

Dental examination was conducted on children by trained dentists, and each caregiver was asked to answer the questionnaire regarding the child’s dental care status, dental care utilization, as well as their attitude and knowledge toward the oral health of their child.

According to Andersen’s behavioral model of healthcare utilization, the independent variables included three characteristics, like predisposing factors, enabling factors, and need factors. In this study, three variables were identified as predisposing factors: (1) region, dichotomously coded as urban and rural; (2) child’s gender, coded as male or female; and (3) caregiver’s gender, coded as male or female. Enabling factors included education status, which was grouped into illiterate, high school, and less than or more than 9 years; family income per year which was categorized into five groups: CNY0–15,000, CNY 15,000–30,000, CNY 30,000–45,000, > CNY 45,000, and missing. Caregiver’s perceived oral health condition of their child was an important need factor. The question stated “what is your opinion about child’s oral health” and the response codes included 5 levels from better to worse. The need factors also include the prevalence of utilization of child’s dental care.

The outcome variable was “How long has it been since your child visited a dentist last time?” and the options were “within six months, six to twelve months, above twelve months, and never” which was answered by their caregivers. We coded the answer dichotomously as yes and no.

The survey was approved by the ethics committee of the College of Stomatology, Xi’an Jiaotong University. All subjects’ rights were protected, and all data was kept confidential.

## Analyses

The data from the questionnaire and oral examination were entered in duplicate to create the database, and the consistency of the two datasets was compared by the EpiData program. Software SPSS version 26 was employed to perform all statistical analyses. The frequencies of variables were calculated by descriptive statistical analysis. Chi-square test was used to evaluate the association between the dental care utilization and the independent variables (caregivers’ gender, education levels, region, caregiver’s self-perceived oral health, overall health about their child, household income, and dental caries). *p* value < 0.05 was considered to be statistically significant. Logistic regression analysis was used to explore the predictors of oral health service utilization independently.

## Results

This study included 492 and 399 caregiver-child pairs in 2015 and 2005 respectively. In 2015, 50.2% of the respondents were from urban areas, while in 2005 47.4% of the respondents belonged to the urban areas. The percentage of male respondents answering the questionnaire was 50.0% in 2015 and 50.1% in 2005. Caregiver’s perception of their child’s oral health was rated as well and very well by 62.2% in 2015 and 48.9% in 2005. The prevalence of dental caries was higher in 2015 (63.2%) than in 2005 (53.4%). Demographic characteristics of subjects such as gender, education level, and dental care utilization are illustrated in Tables 1 and 2.

**Table 1:** Descriptive and bivariate analyses of the independent variables related to the subjects in the study

Characteristics	Variables	2015	%	2005	%
*Predisposing factors*					
Region	Urban	247	50.2	189	47.4
	Rural	245	49.8	210	52.6
Child’s gender^*^	Male	246	50.0	200	50.1
	Female	246	50.0	199	49.9
Caregiver’s gender	Male	124	25.2	127	31.8
	Female	368	74.8	272	68.2
*Enabling factors*					
Education level^*^	Illiterate	34	6.9	18	4.5
	High school and below	394	80.1	282	70.7
	Above 9 years	64	13.0	99	24.8
Family income^*^	0–15,000	60	12.2	226	56.6
	15,000–30,000	123	25.0	109	27.3
	30,000–45,000	30	6.1	17	4.3
	≥ 45,000	196	39.8	10	2.5
	Missing	83	16.9	37	9.3
*Need factors*					
Self-rated oral health^*^	Very well	126	25.6	61	15.3
	Well	180	36.6	134	33.6
	Average	140	28.4	151	37.8
	Poor	37	7.5	46	11.5
	Worse	9	1.9	7	1.8
Caries^*^	Yes	311	63.2	213	53.4
	No	181	36.8	186	46.6
Total		492		399	

*p ≤ 0.05

**Table 2:** Oral health services utilization in 2015 and 2005

Variables	2015	%	2005	%
*Your child visited a dentist*				
Yes	98	19.9	83	20.8
No	394	80.1	316	79.2
The reason to see a dentist^*^				
Treatment	45	45.9	33	39.8
Check-up	19	19.4	20	24.1
Prevention	2	2.0	2	2.4
Do not know	32	32.7	28	33.7

*p ≤ 0.05

In single-factor analyses, caries was significantly associated with dental care utilization. Compared with 2015, predisposing factors and enabling factors were crucial for dental service use in 2005, especially the region, caregiver’s gender, level of education, and family income. While in 2015, caregiver’s perceived oral health about their child was statistically significant (Table 3).

**Table 3:** Factors associated with dental care service utilization

Variables		Yes	%	No	%
		2015/2005		2015/2005	
*Predisposing factors*					
Region^**^	Urban	50/58	51.0/69.9	197/131	50.0/41.5
	Rural	48/25	49.9/30.1	197/185	50.5/58.5
Child’s gender	Male	49/41	50.0/49.4	196/159	49.7/50.3
	Female	49/42	50.0/50.6	198/157	50.3/49.7
Caregiver’s gender^**^	Male	23/18	23.5/21.7	101/109	25.6/34.5
	Female	75/65	76.5/78.3	293/207	74.4/65.5
*Enabling factors*					
Education level^**^	Illiterate	6/2	6.1/2.4	28/16	7.2/5.1
	High school and below	79/48	80.6/55.8	315/234	79.9/74.1
	Above 9 years	13/33	13.3/41.8	51/66	12.9/20.8
Family income^**^	0–15,000	14/31	14.3/37.3	46/195	11.7/61.7
	15,000–30,000	20/33	20.4/39.8	103/76	26.1/24.1
	30,000–45,000	6/6	6.1/7.2	24/11	6.1/3.5
	≥ 45,000	44/4	44.9/4.8	152/6	38.6/1.9
	Missing	14/9	14.3/10.9	69/28	17.5/8.8
*Need factors*					
Caries^***^	Yes	88/55	89.8/66.3	223/158	56.6/50.0
	No	10/28	10.2/33.7	171/158	43.4/50.0
Self-rated oral health^*^	Very well	8/7	8.2/8.4	118/54	29.9/17.1
	Well	21/28	21.4/33.7	159/106	40.3/33.5
	Average	42/31	42.9/37.3	98/120	24.9/38.0
	Poor	19/14	19.3/16.9	18/32	4.6/10.1
	Worse	8/3	8.2/3.7	1/4	0.3/1.3

*p ≤ 0.05 significantly association in 2015

**p ≤ 0.05 significantly association in 2005

***p ≤ 0.05 significantly association in 2015 and 2005; *p* values were based on chi-square test

All independent variables were included in the logistic regression analyses was used to study the confounding factors. In 2005, dental care utilization was higher among male, urban caregivers, and among children who have caries. While in 2015, caregivers who had better perceived oral health about their child and child with caries demonstrated increased utilization of oral health service (Table 4).

**Table 4:** Logistic regression analysis of factors possibly associated with dental care utilization of pre-school children in 2005 and 2015

Dimensions	Characteristics	2015	2005
		OR (95%CI)	OR (95%CI)
Predisposing factors	Region (vs. rural)		
	Urban	0.88(0.49–1.56)	0.35(0.19–0.63)^*^
	Child’s gender (vs. female)		
	Male	0.95(0.55–1.65)	1.02(0.58–1.79)
	Caregiver’s gender (vs. female)		
	Male	1.05(0.56–1.97)	2.11(1.08–4.15)^*^
Enabling factors	Education level (vs. Above 9 years)		
	Illiterate	1.41(0.29–6.80)	2.75(0.51–14.82)
	High school and below	1.03(0.45–2.33)	1.28(0.63–2.59)
	Family income (ten thousand per year) (vs. ≥ 4.5)		
	0–1.5	1.00(0.45–2.22)	4.54(1.04–19.93)^*^
	1.5–.30	2.21(1.09–4.49)^*^	2.03(0.47–8.77)
	3.0–4.5	1.18(0.39–3.57)	1.64(0.29–9.77)
	Missing	/	/
Need factors	Self-rated oral health (vs. Worse)		
	Very well	62.03(6.07–634.20)^*^	2.94(0.45–19.22)
	Well	36.38(3.84–344.29)^*^	1.64(0.30–8.97)
	Average	11.04(1.20–101.27)^*^	1.80(0.33–9.90)
	Poor	6.82(0.68–68.31)	1.20(0.21–7.04)
	Caries (vs. no)		
	Yes	0.21(0.10–0.43) ^*^	− 0.42(0.22–0.77)^*^

*p ≤ 0.05

## Discussion

In this study, we analyzed the data from two cross-sectional surveys in northwest China to show the empirical relationship between caries, caregiver’s perceived oral health, and oral health service utilization over a decade. The study revealed a few novel findings. We observed that northwestern China has experienced a minor difference in the utilization of dental services among pre-school children over the past decade despite an increase in family income and the availability of more accessible dental care services.

In 2005, we found that few need factors were associated with the utilization of dental care services, which revealed the other potential determinants of oral health use. Urban residence and higher family income, after adjusting other confounding factors, were independently associated with dental care utilization. Moreover, the caregiver’s gender was significantly associated with dental care use. Thus, these results suggest that socio-economic inequalities play a crucial role in oral health service utilization.

Socio-demographic characteristics prevent children from seeking care in 2005, while in 2015, there was no significant difference in the use of dental services among respondents from rural and urban areas. It can be attributed to the fact that rural oral health service resources have gradually improved and become more convenient. Moreover, caregivers are more inclined to seek dental care for their children. Though the family income per year has increased almost 3.5 times, from RMB16037 to RMB54266 over the past decade, the dental care service was still underutilization. The results indicate caregivers need to be encouraged and guided toward the utilization of children's oral preventive health care services. Health promotion activities for parents are imperative to combat the underutilization of oral health services by caregivers. This can be achieved by enhancing the oral health education system through specific strategies targeting excluded populations. This may lead to increased awareness of the child’s oral status and help caregivers transform their demand into a need in the future.

The prevalence of dental care utilization among pre-school children was still low in 2015, though the deciding factors were different from those in 2005. Our finding suggests that need factors play a major role in the use of oral health care in 2015. Caregivers with better conception about their child’s oral health demonstrated higher use of dental service. Studies have shown that caregivers with better knowledge and attitude about oral health are more likely to seek dental care services. In addition, the significantly increased rate of treatment and higher probability of caries indicates the necessity for creating specific education programs.

## Conclusions

We can conclude that over the past ten years, oral health service utilization among pre-school children has been limited in northwest China. Policies like providing appropriate and specific oral health education about pre-school children and their oral health status even in the high-income groups will not only lower the incidence of caries but will also lead to the overall improvement of oral health service utilization.

### Limitation

The factors used in our study are limited, additional variables (e.g., medical insurance, dentist-to-population ratio) should be also explored in future research. In order to reduce the prevalence of caries and improve access to care, culturally acceptable policies and interventions are needed.

## Data Availability

The datasets analyzed in the current study are available from the corresponding author on reasonable request.
